# Effects of oral antidiabetic drugs on left ventricular mass in patients with type 2 diabetes mellitus: a network meta-analysis

**DOI:** 10.1186/s12933-018-0773-1

**Published:** 2018-09-27

**Authors:** Satoshi Ida, Ryutaro Kaneko, Kazuya Murata

**Affiliations:** Department of Diabetes and Metabolism, Ise Red Cross Hospital, 1-471-2, Funae, 1-chome, Ise-shi, Mie 516-8512 Japan

**Keywords:** Antidiabetic drugs, Network meta-analysis, Randomized controlled trials, Type 2 diabetes mellitus, Left ventricular mass

## Abstract

**Background:**

We used a network meta-analysis of randomized controlled trials (RCTs) to comparatively examine the effects of oral antidiabetic drugs (OADs) on left ventricular mass (LVM) in patients with type 2 diabetes.

**Methods:**

Document searches were implemented using Medline, Cochrane Controlled Trials Registry, and ClinicalTrials.gov. We decided to include RCTs that evaluated the impact of LVM using the administration of OADs to patients with type 2 diabetes. The outcome evaluations used standardized mean difference (SMD) and 95% confidence intervals (CIs). We then performed a comparative examination of LVM related to the administration of OADs using random effects network meta-analysis.

**Results:**

The document search found 11 RCTs (1410 people) that satisfied the eligibility criteria for this study, and these RCTs were incorporated into the network meta-analysis. The only medication that significantly reduced LVM compared to a placebo was gliclazide (SMD, −1.09; 95% CI, −1.62 to  − 0.57). Further, when comparing the impact on LVM between OADs, only gliclazide significantly reduced LVM compared to other OADs (glyburide, voglibose, metformin, pioglitazone, rosiglitazone, and sitagliptin).

**Conclusions:**

In the present study, gliclazide was the only medication that significantly reduced LVM in patients with type 2 diabetes. When considered from the perspective of causing heart failure and preventing recurrence, it is possible that the use of gliclazide in patients with type 2 diabetes will provide multiple benefits.

## Background

Cardiovascular disease in patients with type 2 diabetes is linked to increased risk of death, which is an extremely important clinical outcome [1]. In recent years, an increase in heart failure among patients with type 2 diabetes has become a grave issue, and the prevention and management of heart disease has become an important focus [2]. Further, type 2 diabetes is clearly an independent risk factor in the occurrence and progress of heart failure [3]. According to previous research, there are several individuals with type 2 diabetes with increased left ventricular mass (LVM) [4–6]. It is believed that increased LVM is linked to microvascular disease, inflammation, and increased oxidative stress. In addition, it is associated with increased insulin resistance, myocardial fibrosis, and left ventricular remodeling because of chronic high blood sugar [7–9]. Increased LVM is a strong predictive factor in the occurrence of cardiovascular diseases such as heart failure, sudden death, and death [10, 11]. It has also been identified as a possible early marker for left ventricular diastolic dysfunction [12]. Consequently, it is believed that in type 2 diabetes, an increased LVM is a problem in clinical practice that needs to be addressed.

Oral antidiabetic drugs (OADs) for patients with type 2 diabetes decrease blood glucose level through increased insulin sensitivity or accelerated insulin secretions. Consequently, several OADs also have the following effects: anti-inflammatory, anti-oxidation, vascular protection, and suppression of myocardial fibrosis. They are also thought to possibly reduce LVM [2, 13]. Previous research has shown LVM reduction though the administration of sulfonylureas [14], thiazolidines [15], or dipeptidyl peptidase 4(DPP4) blockers [16]. Nevertheless, some reports have also shown no significant LVM reduction upon the administration of OADs [17–19] and inconsistent effects.

Previous research includes reports of randomized controlled trials (RCTs) concerning the effect of OADs on LVM when administered to patients with type 2 diabetes. However, reported RCTs of drug effects are limited, and at several instances, comparative results regarding the effects of target drugs cannot be evaluated. Therefore, based on existing RCTs, we believe that a network meta-analysis that is capable of indirectly comparing effects between drugs would be useful. The purpose of this research is to use RCT network meta-analysis to examine the impact of the administration of OADs on LVM in patients with type 2 diabetes.

## Methods

### Study selection

A document search was performed using Medline, Cochrane Controlled Trials Registry, and ClinicalTrials.gov (January 1, 2018). The search strategy was implemented by multiplying the search formulas for type 2 diabetes, OADs, and RCTs (Appendix 1). RCTs that evaluated the impact on LVM of OADs administered to patients with type 2 diabetes were included in this study. Regardless of whether medical diets or exercise therapy were used, tests that comparatively examined the impact on LVM between OADs and a placebo, or between OADs were implemented. Exclusion criteria included the following: animal experimentation, research that was not an RCT, research targeting gestational diabetes, research with insufficient data despite analysis being performed, and duplicate documents. Two authors (SI and RK) independently evaluated whether each document satisfied the eligibility requirements for this research. If they disagreed in their interpretation, they consulted a third reviewer (KM).

### Data extraction and quality assessment

A data extraction form, describing research characteristics, was included in this study (key author’s name, publication year, study location, sample size, patient’s baseline information, basic treatment, and treatment duration). We included the mean, standard deviation, and standard error or 95% confidence intervals (CIs) for LVM, which was the outcome. If trials compared multiple intervention groups with the same control group within one comparison, the shared control group was considered as two or more groups. Two authors (SI and RK) independently evaluated the quality of research that was included in the present study using Cochran’s risk of bias tool [20]. Evaluation used low risk of bias, moderate risk of bias, or high risk of bias in six domains (random sequence generation, allocation concealment, blinding of personnel and participants, blinding of outcome assessors, incomplete data, and selective reporting).

### Statistical analysis

LVM was a continuous variable, and it was predicted that each research study would be described using different units, so our analysis used standardized mean difference (SMD) and 95% CIs. The effectiveness of treatment was the difference between the groups in the amount of LVM change before and after treatment. If only the standard error or *P*-values were described, standard deviation was calculated as described by Altman and Bland [21]. If no standard deviation was described, standard deviation was calculated from 95% Cis, *t*-values, or *P*-values [22].

First, we performed a standard pairwise meta-analysis using a random effects model as a direct comparison. Next, we performed a network meta-analysis as an indirect comparison. The random effects network meta-analysis was performed using mvmeta routine in STATA 13 statistical software (StataCorp. College Station, Texas, USA) [23, 24], and the evidence from direct and indirect comparisons was merged. In addition, we also examined the treatment hierarchy using a Surface Under the Cumulative RAnking curve (SUCRA). SUCRA is an index that estimates in percentage order which treatments are most useful for outcomes [25]. The closer SUCRA was to 100, the more useful the treatment, and results tending toward 0 indicated poor.

We used the following methods to assess any inconsistencies between direct and indirect comparisons. First, we evaluated whether there were any local inconsistencies by comparing treatment effects in the direct and indirect comparisons using all closed loops on the network (loop-specific test) [25]. Next, we looked for any global inconsistencies by evaluating the agreements of evidence obtained from different treatment designs to see if there were any inconsistencies in the overall network (A design-by-treatment interaction model) [26]. If the P value of the test results for local and global inconsistencies was 0.05 or greater, it was judged that there were no inconsistencies in the results of the direct and indirect comparisons.

## Results

### Description of included studies

**Fig. 1:**
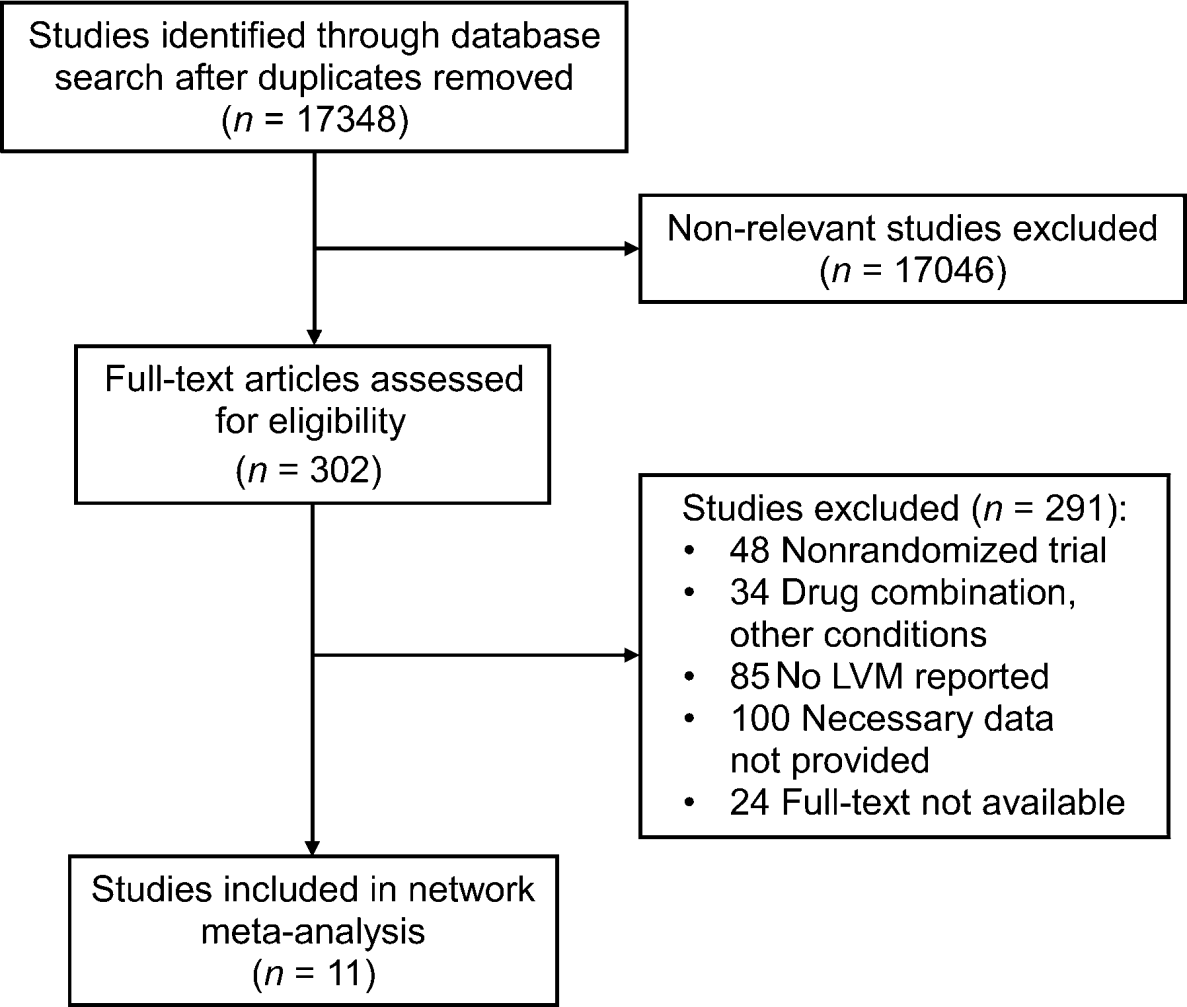
Study flow diagram. *LVM* left ventricular mass

**Fig. 2:**
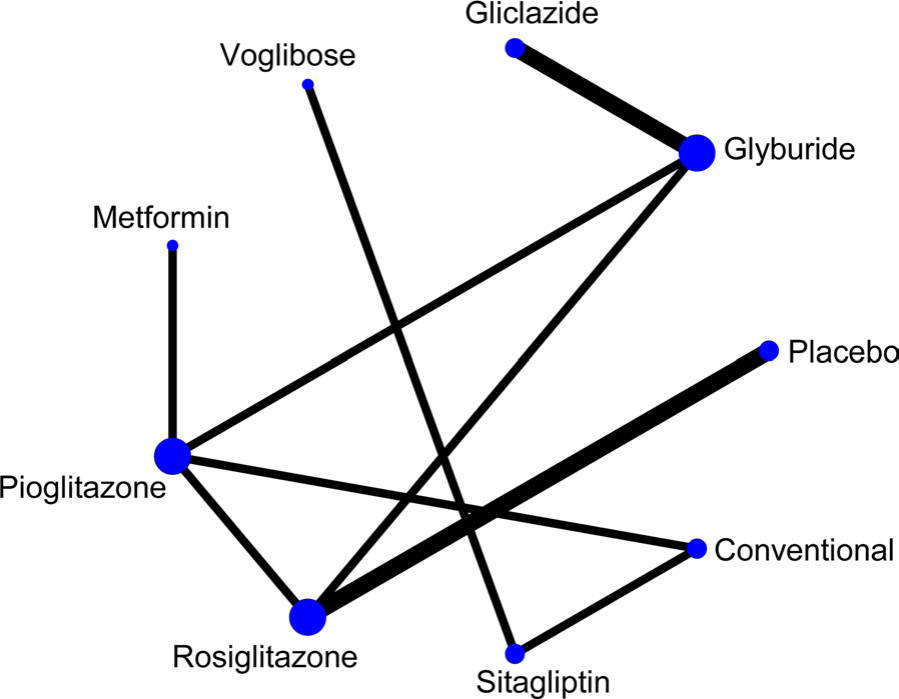
Network of clinical trials on oral antidiabetic drugs or placebo in patients with type 2 diabetes. Lines connect the interventions that have been studied in head-to-head comparisons in eligible RCTs. The width of the lines represents the total number of RCTs for each pairwise comparison. The size of every node is proportional to the number of randomized participants

Document search retrieved 17,348 papers and 11 RCTs (1410 individuals) that matched the eligibility criteria for this study. These findings were included in the meta-analysis (Fig. 1) [14, 17–19, 27–33]. Features of the 11 RCTs are shown in Table 1, and the network map is shown in Fig. 2. Age of the target patients was 60.3 years, and 44.6% of the patients were women. The average time since diagnosis of diabetes was 8.4 years, and average trial period was 32.3 weeks. Seven types of oral diabetes medication (glyburide, gliclazide, voglibose, metformin, pioglitazone, rosiglitazone, and sitagliptin) and a placebo were included in the analysis.

**Table 1:** Characteristics of the studies included in the network meta-analysis

No.	References	Year	Region	No. of patients	Age (years)	% Women	BMI (kg/m^2^)	Body weight (kg)	Duration of DM (years)	HbA1c (%)	Hypertension (%)	Dyslipidemia (%)	Prior CVD (%)	Comparison	OADs dose (mg/day)	Basic treatment	Study duration (weeks)	LVM (g) or LVMI (g/m^2^)
1	Yamada et al. [17]	2017	Japan	115	69	35	24.8	NR	NR	6.9	76	70	0	Sitagliptin vs. conventional	Sitagliptin, 25 or 50; conventional, α-glucosidase inhibitor/glinide/metformin/sulfonylurea/pioglitazone	Diet + exercise	96	96.2 (g/m^2^)
2	Oe et al. [18]	2015	Japan	77	66	35	25.7	NR	3	NR	80	10	5	Sitagliptin vs. voglibose	Sitagliptin,50; voglibose, 0.6	Diet + exercise	24	85 (g/m^2^)
3	McGavock et al. [27]	2012	US	49	55	52	34	92	10.7	7.7	78	65	8	Rosiglitazone vs. placebo	Rosiglitazone, 8	Diet + exercise	24	153 (g)
4	Naka et al. [28]	2010	Greece	81	64	72	NR	74.2	9	7.9	NR	NR	0	Pioglitazone vs. conventional	Pioglitazone, 30; conventional, metformin/sulfonylurea	Metformin and/or sulfonylurea	24	118.1 (g/m^2^)
5	McGuire et al. [29]	2010	US	108	55	38	34	97	8.7	7.2	74	75	37	Rosiglitazone vs. placebo	Rosiglitazone, 8	Diet + exercise	24	76 (g/m^2^)
6	Pala et al. [30]	2010	Turkey	40	55	60	33	NR	4.4	8.4	65	60	0	Rosiglitazone vs. pioglitazone	Rosiglitazone, 8; pioglitazone, 30	Metformin and/or sulfonylurea	16	136 (g/m^2^)
7	van der Meer et al. [19]	2009	Netherlands	71	56	NR	29.3	NR	NR	7	NR	NR	0	Pioglitazone vs. metformin	Pioglitazone, 30; metformin, 2000	Diet + exercise	24	107 (g)
8	Giles et al. [31]	2008	US	518	63	33	29.7	NR	11.6	8.9	NR	NR	100	Pioglitazone vs. glyburide	Pioglitazone, 30; glyburide, 10	Metformin and/or sulfonylurea	24	NR
9	Lee et al. [14].	2007	Taiwan	108	63	44	26.6	NR	11	8.3	74	NR	0	Glyburide vs. gliclazide	Glyburide, 5; gliclazide, 80	Diet + exercise	24	219 (g)
10	Pan et al. [32]	2006	Taiwan	40	63	52	27	NR	12	8.1	76	NR	0	Glibenclamide vs. gliclazide	Glibenclamide, 5; gliclazide, 80	Glibenclamide	24	120 (g/m^2^)
11	Sutton et al. [33]	2002	US	203	55	25	NR	86.2	5.3	9.1	NR	NR	0	Glyburide vs. rosiglitazone	Glyburide, 10; rosiglitazone, 8	Diet + exercise	52	75.5 (g/m^2^)

Unless indicated otherwise, data are shown as mean values

*DM* diabetes mellitus, *BMI* body mass index, *OADs* oral antidiabetic drugs, *LVM* left ventricular mass, *NR* not reported

### Assessment of potential bias

The percentage of suitable descriptions by domain were as follows: random sequence generation was 45.4% (5/11), allocation concealment was 45.4% (5/11), blinding of participants and personnel was 36.3% (4/11), blinding of outcome assessors was 45.4% (5/11), incomplete data was 36.3% (4/11), and selective reporting was 90.9% (10/11) (Table 2). Variation in the quality of the included RCTs was high. Altogether, the overall risk of bias was high.

**Table 2:** Risk of bias assessment included in the network meta-analysis

No.	Reference	Randomization procedure	Allocation concealment	Blinding of personnel and participants	Blinding of outcome assessment	Incomplete outcome assessment	Selective reporting
1	Yamada et al. [17]	L	L	H	L	L	L
2	Oe et al. [18]	L	L	H	U	U	L
3	McGavock et al. [27]	L	L	L	L	U	L
4	Naka et al. [28]	L	L	H	L	U	L
5	McGuireet al. [29]	U	U	L	U	H	L
6	Pala et al. [30]	U	U	U	U	L	L
7	van der Meer et al. [19]	L	L	L	U	L	L
8	Giles et al. [31]	U	U	L	U	U	L
9	Pan et al. [32]	U	U	U	L	L	L
10	Sutton et al. [33]	U	U	H	L	U	L
11	Lee et al. [14]	U	U	H	L	H	L

*L* low risk of bias, *U* unclear risk of bias, *H* high risk of bias

### Direct pairwise meta-analysis

Table 3 shows the results of the direct pairwise meta-analysis. One RCT compared OADs and a placebo in terms of the effect on LVM (rosiglitazone vs. placebo), finding no statistically significant difference. Alternatively, among the studies that compared the effects on LVM between OADs, the only significant difference that was identified was in the comparative trials between glyburide and gliclazide (SMD, −0.95; 95% CI, −1.29 to  − 0.61), where the gliclazide cohort showed a significant decrease in LVM compared to the glyburide cohort.

**Table 3:** Results of network meta-analysis (data under the cells marked with italic drugs) and direct comparison (data above the cells marked with italic drugs) of all treatments

*Placebo*						− 0.05 (− 0.36, 0.27) [27, 29]		
0.14 (− 0.26, 0.54)	*Glyburide*	− 0.95 (− 1.29, − 0.61) [14, 32]			0.07 (− 0.11, 0.24) [31]	0.09 (− 0.19, 0.36) [33]		
1.09 (0.57, 1.62)	0.95 (0.61, 1.29)	*Gliclazide*						
0.12 (− 0.72, 0.96)	− 0.02 (− 0.76, 0.73)	− 0.97 (− 1.79, − 0.15)	*Voglibose*				− 0.20 (− 0.65, 0.25) [18]	
0.13 (− 0.50, 0.76)	− 0.01 (− 0.50, 0.49)	− 0.96 (− 1.56, − 0.36)	0.01 (− 0.85, 0.87)	*Metformin*	0.05 (− 0.41, 0.52) [19]			
0.08 (− 0.35, 0.50)	− 0.06 (− 0.23, 0.10)	− 1.02 (− 1.40, − 0.64)	− 0.05 (− 0.77, 0.68)	− 0.05 (− 0.52, 0.41)	*Pioglitazone*	0.06 (− 0.56, 0.68) [30]		− 0.06 (− 0.49, 0.38) [28]
0.05 (− 0.27, 0.36)	− 0.10 (− 0.35, 0.16)	− 1.05 (− 1.47, − 0.62)	− 0.08 (− 0.86, 0.70)	− − 0.09 (− 0.63, 0.46)	− 0.03 (− 0.32, 0.26)	*Rosiglitazone*		
0.32 (− 0.39, 1.03)	0.18 (− 0.41, 0.77)	− 0.77 (− 1.46, − 0.09)	0.20 (− 0.25, 0.65)	0.19 (− 0.55, 0.92)	0.24 (− 0.33, 0.81)	0.27 (− 0.36, 0.91)	*Sitagliptin*	0.18 (− 0.18, 0.55) [17]
0.14 (− 0.47, 0.74)	− 0.01 (− 0.47, 0.46)	− 0.96 (− 1.54, − 0.38)	0.01 (− 0.57, 0.59)	0.00 (− 0.63, 0.64)	0.06 (− 0.38, 0.49)	0.09 (− 0.43, 0.61)	− 0.18 (− 0.55, 0.18)	*Conventional*

### Network meta-analysis

Table 3 shows the results of the network meta-analysis. The only medication that showed a significant difference in reducing LVM compared to the placebo was gliclazide (SMD, −1.09; 95% CI, −1.62 to  − 0.57). Further, when we examined the impact on LVM between OADs, only gliclazide significantly reduced LVM compared to other OADs. Table 4 shows the results of the SUCRA analysis. The drug with the highest SUCRA values was gliclazide (99.6%), followed by sitagliptin (68.8%). The placebo has the lowest SUCRA values (28.1%).

**Table 4:** The rank of oral antidiabetic drugs on left ventricular mass

Treatment	SUCRA	Rank
Placebo	28.1	9
Glyburide	51.3	3
Gliclazide	99.6	1
Voglibose	43.3	6
Metformin	45.2	4
Pioglitazone	36.4	7
Rosiglitazone	32.8	8
Sitagliptin	68.8	2
Conventional	44.4	5

*SUCRA* Surface Under the Cumulative RAnking curve

### Inconsistency between direct and indirect evidence

Only one closed loop (triangular loop: glyburide–pioglitazone–rosiglitazone) was found regarding local inconsistencies. There was no significant difference in the loop-specific test, which was consistent (P = 0.913). No significant inconsistencies were identified between the direct and indirect comparisons using the design-by-treatment interaction model for global inconsistencies (P = 0.913).

## Discussion

A significant number of patients have increased LVM among those with type 2 diabetes [4, 34, 35]. It is believed that the mechanism of increased LVM is related to microvascular disease, inflammation, obesity, elevated oxidative stress, increased insulin resistance, myocardial fibrosis, left ventricular remodeling, and other conditions [7–9]. Meanwhile, as increased LVM and impaired diastolic dysfunction are believed to impair glucose tolerance, poor blood glucose management and increased LVM seem to closely correlate with each other [36]. High LVM is a strong predictive factor in the occurrence of cardiovascular disease beginning with heart failure and progressing to death [10, 37]. Consequently, it is believed that increased LVM is a clinical problem in type 2 diabetes. In this study, we indirectly compare type 2 diabetes through network meta-analysis. As a result, only gliclazide significantly reduces LVM compared to the placebo and other OADs. It has been found in a previous study that sulfonylureas bond to sulfonylurea receptors (SUR) in the pancreatic β cell membrane; thereby, causing insulin secretion [38]. Furthermore, it has also been reported that sulfonylureas act outside the pancreas in addition to the action of lowering blood sugar due to the stimulus of insulin secretion. Among the drugs being studied, gliclazide is thought to have strong anti-oxidation and anti-inflammatory effects derived from the azabicyclo-octyl ring in its structure [39]. As aforementioned, inflammation and elevated oxidative stress levels are closely associated with left ventricular remodeling and increased LVM [7–9, 40, 41]. It appears that the inhibitory action of gliclazide on oxidative stress and inflammation is the mechanism by which LVM is reduced. Moreover, in addition to being expressed from pancreatic beta cells, it has been found that SUR are expressed on the surface of myocardial cells [42]. It is thought that closing the ATP receptor K^+^ channel by bonding to SUR in the myocardial cells and increasing endothelin-1 (ET-1) are possibly involved with elevated LVM [15]. Gliclazide has high SUR selectivity in pancreatic β cells; thus, its action on SUR in myocardial cells is thought to be minimal [42]. This is believed to be the reason why gliclazide significantly lowers LVM compared to glyburide, despite both being sulfonylureas.

However, except gliclazide, no OADs exhibited significant LVM-lowering effects. In a previous study on patients with type 2 diabetes, it was reported that thiazolidine derivatives reduced LVM more than other administered drugs [15]. However, it has also been reported that thiazolidine derivatives do not have LVM-lowering or cardioprotective effect [28, 43]. In an animal experiment, DPP4 inhibitors reduced LVM more than vildagliptin [16], and it has been indicated that the administration of incretin preparations has anti-inflammatory and LVM-lowering actions [44, 45]. While metformin is believed to exhibit anti-inflammatory and anti-oxidative actions, it has been reported that no LVM-lowering effect was observed [19, 46]. In the present study, while the administration of these drugs did not lower LVM significantly compared with the placebo, the results lack consistency with those of previous studies, and we believe that further examination is warranted.

Our research is the first report to examine how administering OADs to patients with type 2 diabetes impacts LVM using the network meta-analysis method. By indirect comparisons using a network meta-analysis, we can verify the effects on LVM by seven different OADs and a placebo. Interestingly, gliclazide was administered to all the participants in the therapeutic intensification cohort in Action in Diabetes and Vascular Disease (ADVANCE) research, and among this cohort, there was little occurrence of cardiovascular disease [47]. Moreover, there are also reports that administering gliclazide to patients with type 2 diabetes decreases the number of cardiovascular deaths [48]. Conversely, in the Action to Control Cardiovascular Risk in Diabetes research, the therapeutic intensification cohort was administered drugs other than gliclazide, and no suppression of cardiovascular disease occurrence was observed in this group [49]. It is possible that gliclazide is beneficial to patients with type 2 diabetes. However, further examination is required for determining whether or not gliclazide therapy reduced mortality by reducing LVM. Furthermore, when using antidiabetic drugs, both the risks and benefits need to be taken into consideration. While gliclazide is believed to have a relatively low risk of hypoglycemia among sulfonylureas, attention should be paid to the risk of hypoglycemia.

### Limitations

Our study has several limitations. First, comparatively, few RCTs are included in this study, and it is possible that due to a lack of manpower, our detection abilities were hampered. Second, it is possible that there are relevant documents in databases that have not been searched that could affect the results. Third, among the RCTs included in the present study, a great discrepancy was noted between each study in terms of the observation period, LVM evaluation method (echocardiography and magnetic resonance imaging), the prevalence of cardiovascular disease, and the drug dosage used. Consequently, caution is required when interpreting the results and generalizing our findings. Fourth, the quality of the RCTs included in this study is generally low; consequently, we have some hesitation about the validity of the research results. Finally, the RCTs included in this study do not include sodium glucose cotransporter 2 (SGLT2) blockers and glinides, so their impact on LVM remains unclear. In particular, with regards to SGLT2 inhibitors, it has been reported that the administration of these drugs might inhibit myocardial fibrosis and reduce cardiac size [50]. In patients with type 2 diabetes, we believe that it is important to conduct further studies with regards to the effect of OADs, including SGLT2 inhibitors, on LVM.

## Conclusion

This research evaluates the impact of OADs on LVM among patients with type 2 diabetes using a network meta-analysis. Only gliclazide significantly reduces LVM compared to a placebo and other OADs. As stated above, however, there is little incorporated research, and the overall quality of the research is poor, so caution is required when analyzing these research results. In the future, re-examination is needed with more RCTs included in the meta-analysis, and further research should be conducted to investigate whether lowering LVM will inhibit the onset of heart failure.

## Appendix 1

**Table:**

PubMed	
#1	Diabetes mellitus or diabetes or NIDDM or non-insulin dependent or type 2 diabetes mellitus
#2	Gliclazide or glibenclamide or glimepiride or sulfonylurea or pioglitazone or thiazolidine or thiazolidinediones or sodium glucose co-transporter 2 inhibitor or sodium glucose co-transporter 2 or sodium glucose co-transporter 2 or ipragliflozin or dapagliflozin or luseogliflozin or tofogliflozin or canagliflozin or empagliflozin or biguanides or metformin or acarbose or voglibose or miglitol or α-glucosidase inhibitor or α glucosidase inhibitor or mitiglinide or repaglinide or nateglinide or glinide or incretin or incretins dipeptidyl peptidase 4 Inhibitors or dipeptidyl peptidase 4 inhibitors or saxagliptin or alogliptin or linagliptin or vildagliptin or sitagliptin or teneligliptin or anagliptin or trelagliptin or omarigliptin or antidiabetic drugs or hypoglycemic medications or hypoglycemic agents
#3	“Randomized Controlled Trial” [Publication Type] or “Controlled Clinical Trial” [Publication Type] or Randomized [tiab] or Randomised [tiab] or placebo [tiab] or randomly [tiab]
#4	#1 and #2 and #3
The Cochrane Controlled Trials Registry	
#1	Diabetes mellitus or diabetes or NIDDM or non-insulin dependent or type 2 diabetes mellitus
#2	Gliclazide or glibenclamide or glimepiride or sulfonylurea or pioglitazone or thiazolidine or thiazolidinediones or sodium glucose co-transporter 2 inhibitor or sodium glucose co-transporter 2 or sodium glucose co-transporter 2 or ipragliflozin or dapagliflozin or luseogliflozin or tofogliflozin or canagliflozin or empagliflozin or biguanides or metformin or acarbose or voglibose or miglitol or α-glucosidase inhibitor or α glucosidase inhibitor or mitiglinide or repaglinide or nateglinide or glinide or incretin or incretins dipeptidyl peptidase 4 Inhibitors or dipeptidyl peptidase 4 inhibitors or saxagliptin or alogliptin or linagliptin or vildagliptin or sitagliptin or teneligliptin or anagliptin or trelagliptin or omarigliptin or antidiabetic drugs or hypoglycemic medications or hypoglycemic agents
#3	#1 and #2

## Abbreviations

CI: confidence intervals
RCT: randomized controlled trials
SMD: standardized mean difference
SUR: sulfonylurea receptors
OAD: oral antidiabetic drugs
LVM: left ventricular mass
DPP4: dipeptidyl peptidase 4
SUCRA: Surface Under the Cumulative RAnking curve
ER-1: endothelin-1
ADVANCE: Action in Diabetes and Vascular Disease
SGLT2: sodium glucose cotransporter 2
